# A Retrospective Study of Clinical and Histopathological Features of 81 Cases of Canine Apocrine Gland Adenocarcinoma of the Anal Sac: Independent Clinical and Histopathological Risk Factors Associated with Outcome

**DOI:** 10.3390/ani11113327

**Published:** 2021-11-22

**Authors:** Hannah Wong, Stephanie Byrne, Roberta Rasotto, Randi Drees, Angela Taylor, Simon L. Priestnall, Chiara Leo

**Affiliations:** 1 Department of Pathobiology and Population Sciences, Royal Veterinary College, Hertfordshire AL9 7TA, UK; spriestnall@rvc.ac.uk; 2 Department of Clinical Sciences and Services, Royal Veterinary College, Hertfordshire AL9 7TA, UK; sbyrne@lvvsc.com (S.B.); rdrees@rvc.ac.uk (R.D.); ataylor@rvc.ac.uk (A.T.); chiara.leo@anicura.it (C.L.); 3 Dick White Referrals, Station Farm, Six Mile Bottom, Cambridgeshire CB8 OUH, UK; rr@dwr.co.uk

**Keywords:** anal sac adenocarcinoma, apocrine gland, histology, hypercalcemia, necrosis, prognosis, radiotherapy, surgery, vascular invasion

## Abstract

**Simple Summary:**

Dogs can be affected by a tumour arising from the glands of the anal sacs. These tumours can grow aggressively and spread to other organs, resulting in disruption of bowel movements and/or organ function, ultimately leading to death or euthanasia. The reason why some tumours grow or spread more quickly is not well understood, and veterinarians are not able to distinguish between dogs likely to have better or worse survival times at the time of initial diagnosis. This study evaluates clinical, imaging, and tumour features of dogs with anal sac tumours and examines which features were linked to improved survival times. Smaller tumour size, smaller lymph node size, surgical removal, and treatment with radiotherapy were associated with better survival times. When examining the tumour tissue, the presence of closely packed tumour cells, dead tumour tissue, and invasion of vessels by tumour cells were each associated with worse survival times. Features that impacted survival were used to create two checklists that, when applied to an individual case, may indicate prognosis and therefore help clinicians and owners make more informed clinical decisions.

**Abstract:**

Canine apocrine gland anal sac adenocarcinoma (AGASAC) is a malignant tumour with variable clinical progression. The objective of this study was to use robust multivariate models, based on models employed in human medical oncology, to establish clinical and histopathological risk factors of poor survival. Clinical data and imaging of 81 cases with AGASAC were reviewed. Tissue was available for histological review and immunohistochemistry in 49 cases. Tumour and lymph node size were determined using the response evaluation criteria in the solid tumours system (RECIST). Modelling revealed tumour size over 2 cm, lymph node size grouped in three tiers by the two thresholds 1.6 cm and 5 cm, surgical management, and radiotherapy were independent clinical variables associated with survival, irrespective of tumour stage. Tumour size over 1.3 cm and presence of distant metastasis were independent clinical variables associated with the first progression-free interval. The presence of the histopathological variables of tumour necrosis, a solid histological pattern, and vascular invasion in the primary tumour were independent risk factors of poor survival. Based upon these independent risk factors, scoring algorithms to predict survival in AGASAC patients are presented.

## 1. Introduction

Canine apocrine gland adenocarcinoma of the anal sac (AGASAC) is a malignant epithelial tumour arising from the glands of the anal sac [1,2]. A genetic and breed predisposition for English Cocker Spaniels has been identified; however, sporadic disease occurs across all breeds [3,4]. Metastasis is frequent, often affecting the locoregional lymph nodes. Distant spread occurs widely and can involve the abdominal and thoracic viscera and bone [2,5,6,7]. Paraneoplastic hypercalcaemia, through the effect of tumour-produced parathyroid-hormone-related polypeptide [8,9,10], is present in 25–51% of dogs at diagnosis and can result in life-threatening complications [5]. Reported metastatic rates at diagnosis and overall survival times are variable, ranging from 26% [11] to 79% [5], and 212 days [2] to 1237 days [11], respectively, depending upon the clinical stage and treatment received [2,5,12,13,14,15,16,17,18,19]. Multimodal therapy involving two or more treatment modalities, including surgery, radiation therapy, and/or medical therapy, is typically recommended. Surgery is considered the basis of treatment for non-metastatic AGASAC and AGASAC with regional lymph node metastasis [2,12,20,21]. Radiotherapy and a range of medical agents have been used both as adjuvant or palliative therapy [2,13,14,15,20,22,23,24,25,26,27,28]. Successful management of the primary tumour and metastatic lesions can lead to prolonged survival ranging from 713 to 1035 days [14,18,21,24], in comparison with patients managed solely medically, which has a reported median survival time of 212 days [2]. However, there are contrasting reports on the effect of treatment protocols on outcomes of AGASAC patients [2,5,15,19,22,23], and as yet, a consensus therapeutic approach has not been defined.

Several clinical and histological factors have been suggested as indicators of poor survival or reduced progression-free interval (PFI). Primary tumour size has the greatest volume of evidence, with larger tumours associated with shorter survival times [2,7,15]. Lymph node involvement and distant metastasis have also been reported to have prognostic relevance [2,5,7,15,19]. The influence of hypercalcaemia on survival is ambiguous, with evidence both for and against an association [2,5,7,29]. Studies investigating clinical factors as indicators of PFI are less numerous; however, tumour size, lymph node metastasis [15], sublumbar lymphadenopathy [19], and hypercalcaemia [29] have been associated with a decrease in median PFI (MPFI). The histological variables of necrosis, solid tumour pattern, vascular invasion and peripheral infiltration, when considered in isolation, have been associated with decreased overall survival [11,16,30]. Previous studies have found no association between mitotic count and survival and/or metastasis at presentation [16,31]. The role of the proliferation marker Ki67 in predicting the progression of AGASAC cases has not been clearly defined; however, studies so far have found no association between per cent of Ki67 immunolabelling within the primary tumour and metastatic lymph nodes with overall median survival time (MST) [17,30]. 

In this study, multivariate models optimised for stability were used to establish clinical and histopathological risk factors of decreased survival and PFI in AGASAC patients. Our aim was to provide clinicians treating dogs with AGASAC with replicable algorithms using readily available clinical and pathological data to generate estimated survival times and relative prognosis for the purpose of informing treatment decisions. Guiding that aim were two hypotheses. Firstly, as our case population employed predominant use of CT imaging for patient staging that allowed precise surgical planning for primary and metastatic disease, we hypothesised that surgery would benefit AGASAC patients irrespective of clinically defined disease stage. Secondly, we also hypothesised that histological features associated with decreased survival have additive risk and that the presence of more than one such feature has greater predictive power for decreased survival than a single variable alone.

## 2. Materials and Methods

### 2.1. Selection Criteria

Medical records from a single veterinary referral hospital, the Royal Veterinary College, were searched for dogs diagnosed with AGASAC from 2006 to 2015. Cases were included if they were diagnosed by cytology or histopathology, underwent clinical staging with diagnostic imaging, and had follow-up with at least one hospital visit or documented communication with the referring veterinary practice.

### 2.2. Data Collection

Relevant clinical information was retrieved from the Queen Mother Hospital database at the Royal Veterinary College, including patient breed, age, sex, primary clinical sign, location of mass, presence of palpable sublumbar lymphadenopathy, treatment, and complications. Diagnostic imaging data for each case were reviewed by a board-certified radiologist, RD. Diagnostic imaging included thoracoabdominal computed tomography (CT), thoracic radiographs, and abdominal ultrasound, or combination thereof, as determined by clinician discretion and owner consent. Tumour and lymph node measurements were performed using the response evaluation criteria in solid tumours (RECIST) system and were reviewed by a board-certified radiologist, RD [32]. Distant metastasis was defined as any lesion outside the regional lymph node bed. Metastatic lesions were confirmed by cytology or histology when possible. If sampling was not performed, a lesion was presumed metastatic if it persisted or progressed in size on serial imaging, were multiple in number (within the liver or spleen), or the appearance of the lesion was more likely to represent metastasis as determined by a board-certified radiologist. Hypercalcaemia was defined as ionised calcium greater than the reference range (1.12–1.40 mmol/L) or elevated total calcium (reference range 2.18–2.79 mmol/L) with clinical signs of hypercalcaemia. Data citation (https://doi.org/10.5061/dryad.x95x69pgx).

### 2.3. Histological Analysis

Biopsy specimens had secondary blinded histological review by board-certified pathologists where tissue was available. HW and SP reviewed the haematoxylin and eosin-stained slides, with the scoring performed by consensus. RR, blinded to the clinical and histological data, scored the immunohistochemistry. Histological parameters of pattern type, presence of necrosis, and presence of vascular invasion within the primary tumour or histological evidence of metastasis in any tissue were recorded. The mitotic count of the primary tumour, across 2.37 mm^2^ consecutive fields in the area of highest mitotic activity, was recorded. Histological pattern was defined as the predominant pattern and was categorised as rosette, tubular, or solid. The presence of histological necrosis was defined as any amount of intra-tumoural necrosis. 

### 2.4. Immunohistochemistry

Ki67 immunohistochemical labelling was performed on fresh-cut tissue sections using a 1:150 concentration of monoclonal anti-Ki67 antibody (MIB-1; Dako, Ely, UK) on a BondMax Autostainer Leica, Milton Keynes). Antigen retrieval was at Ph 9 (Bond ER2, Leica, Milton Keynes, UK) and 90 °C for 10 min. The Bond Polymer Redefine Detection kit (Leica) was used for antibody visualisation. A positive reaction was indicated by the presence of specific brown intranuclear immunoreactivity. The positive control was a canine cutaneous mast cell tumour, and this tissue without the Ki67 antibody was used as a negative control. At ×400 magnification, over an area of 2.37 mm^2^, the number of positively immunolabelled Ki67 cells were counted from at least 1000 cells for each sample. Ki67 expression in each sample was defined as the percentage of positive cells relative to the total number of cells counted. 

### 2.5. Statistics

The zero-time point was the date of initial diagnosis confirmed by cytology or histology. Overall survival was defined as the time to death by any cause relatable to AGASAC disease. Cases were censored if the dog was alive at the date of data analysis, had been lost to follow-up, or died due to a defined condition unrelatable to the AGASAC disease. Progression-free interval was defined as the first period from diagnosis until confirmed disease progression on imaging or clinical examination at the referral centre. Cases without documented disease progression were censored at the most recent examination at the referral centre. Cases that did not revisit the referral centre to evaluate progressive disease were not included in the PFI data set. Model variables were identified from review of the literature, and the evidence of effect was considered, with higher-ranking variables included in the global model [33]. Proportional hazard assumptions and randomness of missing data were confirmed. Optimal cut-off values for continuous variables were defined as receiver operating characteristic curve (ROC) values with the highest Youden index [34]. Variance inflation factors were calculated, and the absence of multicollinearity was confirmed. Backwards elimination using multivariate Cox regression with α equal to the Akaike information criterion, 0.157 [35], was used to produce an optimised model [33]. The B coefficients and *p* values were the results of 1000 bootstrap samples. Significant variables were compiled into algorithms. Survival times were calculated using the Kaplan–Meier product-limit method and assessed by Log-rank tests. Hazard ratios were calculated by Cox regression analysis. Statistically significant differences were defined as *p* < 0.05 or using the Bonferroni correction for multiple comparisons. Statistical calculations were performed using SPSS Statistics (version 26, IBM Corp., Armonk, NY, USA) and GraphPad Prism (version 8, GraphPad Software, La Jolla, CA, USA).

## 3. Results

### 3.1. Signalment, Clinical Signs, and Clinical Staging 

Eighty-one cases met the inclusion criteria, and the population signalment is detailed in Table 1.

Thoracoabdominal CT was performed in 61 cases (61/81; 75%). Thoracic radiographs combined with either abdominal ultrasound or abdominal CT was performed in 18 cases (18/81; 22%). Two cases had abdominal ultrasound only (2/81; 2%). Primary disease alone was present in 19 cases (19/81; 23%), 37 cases (37/81; 46%) had nodal metastasis, and 25 cases (25/81; 31%) had distant metastasis. Sixty-five cases had lymphadenopathy (65/81), most commonly of the medial iliac node (n = 49), followed by the hypogastric node (n = 30) and sacral/sublumbar nodes (n = 27). Metastatic disease was ruled out as a cause of the lymphadenopathy in three cases through cytological examination, and in five cases with putative metastatic lesions, owners declined any further investigation. Forty-six cases (46/57; 81%) with putative metastasis that underwent further investigation had metastasis confirmed by cytological or histological examination. Lymph node size was weakly correlated with the presence of hypercalcaemia (Pearson correlation coefficient: 0.313, *p* = 0.006 **). Distant metastases were confirmed either through histology or cytology in the liver (n = 5), spleen (n = 3), pancreas (n = 1), adrenal glands (n = 2), and urinary bladder (n = 1). Lung metastasis (n = 15) was presumed based on CT imaging, except for 1 case in which necropsy was performed. Other presumed metastatic locations included adrenal glands (n = 4), bone (n = 3), vertebral canal (n = 1), intramuscular (n = 3), rectal wall (n = 1), and retroperitoneal (n = 1).

### 3.2. Prognostic Clinical Variables and Patient Survival Time

**Figure 1:**
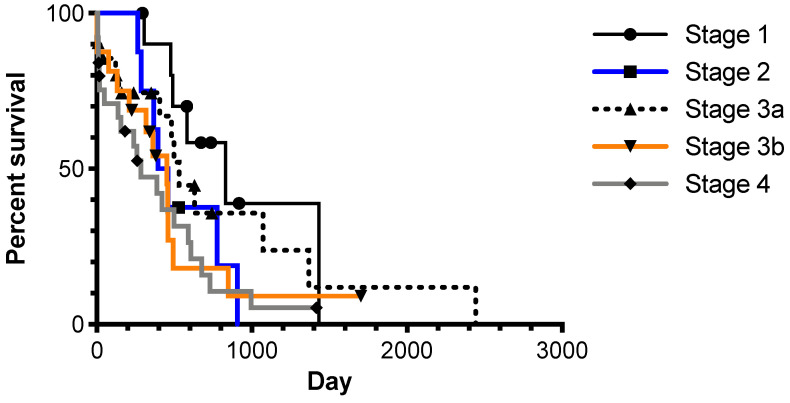
Kaplan–Meier plot of AGASAC cases grouped by Polton stage. Log-rank *p* = 0.1056. Symbols indicate censored cases.

At the time of data analysis, 1 dog was alive, 57 were dead, and 23 were lost to follow-up. A necropsy was performed in one case. The overall MST was 461 days (range 0–2442 days), and the median time to censor was 346 days (range 9–2442 days). Of the 37 dogs with PFI data, 27 dogs had disease progression, and 10 were in remission. The median PFI was 243 days (range 57–686 days), and the median time to censor was 206 days. The median longest tumour diameter was 2.5 cm (range 0.2–10 cm). The median longest lymph node diameter was 2.2 cm (range 0.1–9.6 cm). Specifically, for the twelve dogs presenting with dysuria or tenesmus, all dogs had either a primary tumour >2 cm (n = 10, median 3.8 cm) or a lymph node >5 cm (n = 6, median 7.5 cm). According to the previously suggested clinical grading scheme for AGASAC [7], 12 cases had Stage 1 disease, 9 had Stage 2 disease, 24 had Stage 3a, 11 had Stage 3b, and 26 had Stage 4 disease. Cases classified in different disease stages did not experience significantly different survival times (Figure 1).

Sixty-three dogs underwent surgical management (63/81; 78%), with 37 receiving surgery alone (37/81; 46%), 19 receiving surgery and medical therapy (19/81; 23%), one dog receiving surgery and radiotherapy (1/81; 1%), and six dogs receiving surgery, medical therapy, and radiotherapy (6/81; 7%). Seven dogs received medical therapy without surgery (7/81; 9%), and this included one dog that received radiotherapy alongside medical therapy. Eleven dogs (11/81; 14%) received no further treatment following diagnosis. Surgical approaches consisted of anal sacculectomy, lymphadenectomy, and/or distant metastasectomy. Surgical complications were reported in 14 dogs (14/81; 17%; full details Appendix A). Radiation therapy, used as adjuvant and palliative treatment, included hyperfractionated and hypofractionated protocols, described in Appendix B. Medical therapy was at the clinician’s discretion, resulting in a variety of protocols used. Primary treatment predominantly used carboplatin, melphalan, cyclophosphamide, and/or toceranib as sole agents or in combination (29/32; 91%). Two dogs received chlorambucil, and one dog received mitoxantrone. Rescue medical therapy was used in 17 dogs, implementing the additional agents: doxorubicin, masitinib, pamidronate, and artemisinin (additional details of chemotherapy Appendix C). The majority of hypercalcaemic patients received surgery (14/19; 74%), either alone (7/19; 37%) or in combination with radiation (2/19; 10%) or chemotherapy (8/19; 42%).

The clinical variables included in the multivariate model to investigate prognostic significance on survival in cases of AGASAC were: tumour size, lymph node size, surgical management, radiotherapy, medical therapy, and blood calcium status. Continuous variables were converted to binary variables using the value with the greatest predictive power as defined by ROC curves and Youden Index analysis. Optimal thresholds in relation to survival were a tumour diameter >2 cm, and lymph node diameter stratified using 1.65 cm and 5 cm. To control for an effect of clinical stage on the candidate variables, the model was stratified by clinical stage to control for any stage-dependent changes in baseline hazard effects. Following backwards elimination, the variables that were found to have independent and significant effects upon overall survival irrespective of the clinical stage were tumour diameter >2 cm, lymph node diameter stratified using 1.65 cm and 5 cm, and surgical treatment and radiotherapy (Table 2, full model description in supporting Table S1). Larger tumour and lymph node sizes were positive risk factors for death, and surgical management and radiotherapy (alone or in any combination of treatments) were positive survival factors.

**Figure 2:**
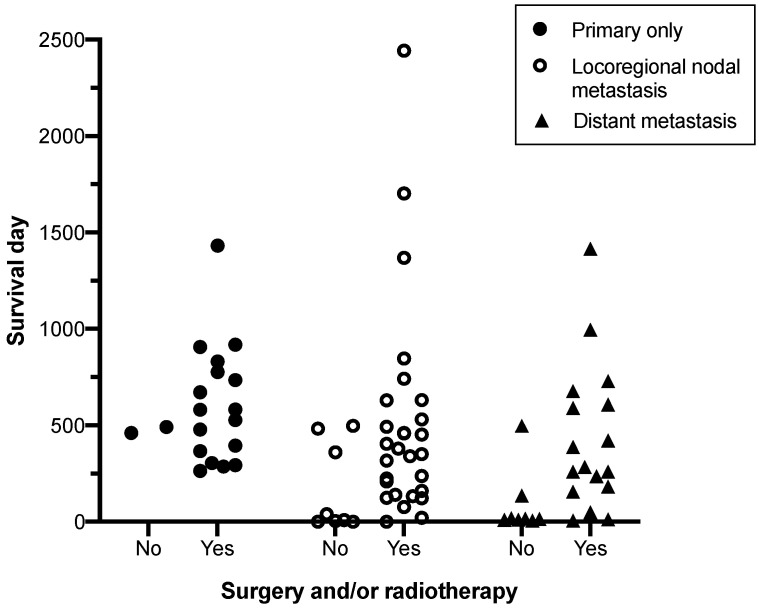
AGASAC patients that underwent aggressive locoregional therapy with surgery and/or radiotherapy had improved survival times compared to patients receiving no treatment or chemotherapy alone. Survival times are grouped by treatment received, and are stratified by clinical stage.

For cases with metastatic disease, including both nodal or distant metastasis, surgical management and the use of radiotherapy, used alone or in combination, resulted in significantly longer MST compared to no treatment or medical treatment alone (Figure 2 and Table 3). Tumour size and lymph node size did not affect surgical or radiotherapy treatment decisions (Supplementary Materials Figure S1).

The variables with a significant independent effect on survival in the multivariate model were combined to form an algorithm, designed to be applied at the point of clinical diagnosis of AGASAC to generate projected survival times based upon clinical features of that individual case and incorporating potential future treatment options (Table 4).

**Figure 3:**
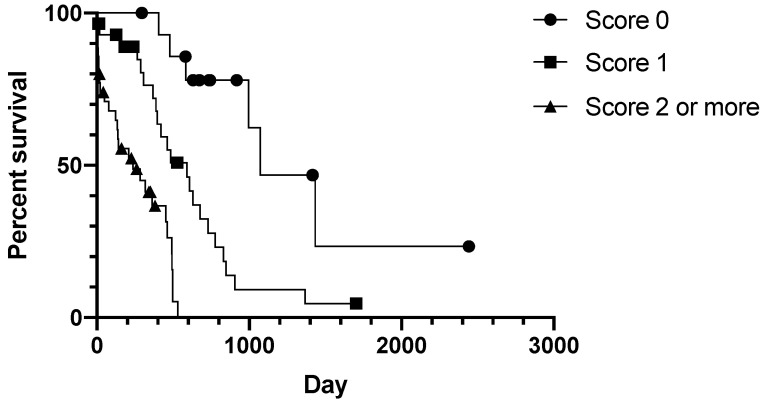
Kaplan–Meier plot of AGASAC cases stratified according to the clinical algorithm detailed in Table 4. Log-rank *p* < 0.0001, all individual curve comparisons *p* < 0.017 (Bonferroni-corrected level of significance).

Applying the clinical algorithm to the study population resulted in three groups with significantly different MST (Log-rank *p* < 0.0001, all individual curve comparisons *p* < 0.017 Bonferroni-corrected level of significance; Figure 3). Cases that scored 0 points (15/77 19%) had an MST of 1072 days (95% CI 666−1659). Cases that scored 1 point (29/77 38%) had an MST of 590 days (95% CI 231−859), and cases that scored 2 points or more (33/77 43%) had an MST of 237 days (95% CI 10−464). Cox regression analysis revealed the groups had a significantly different (*p* = 0.001) hazard ratio of 3.739 (full Cox regression in Supplementary Materials, Table S2).

Following the identification of clinical variables significantly associated with overall survival, the effect of clinical variables on the PFI of cases of AGASAC was also investigated. For PFI, the following variables formed the global model: tumour size, lymph node size, distant metastasis, and hypercalcaemia. Optimised thresholds for tumour and lymph node diameter as predictors of PFI were calculated as 1.3 cm for tumour diameter and 3.8 cm for lymph node diameter. The optimised model revealed that tumour size > 1.3 cm and the presence of distant metastasis were both independently associated with an increased risk of shorter PFI (Table 5; Supplementary Materials Table S3).

### 3.3. Prognostic Histopathological Parameters

Forty-nine cases had tissue available for secondary blinded histological review, and all cases were excisional biopsies. The published evidence from univariate analysis in other studies suggests that the presence of necrosis, vascular invasion, and a solid pattern are associated with decreased survival times, but these factors have not yet been tested using multivariate analysis. In addition, given that mitotic index has been poorly correlated to survival [16], we hypothesised that Ki67 might be a better measure of the proliferative activity in primary AGASAC. 

**Figure 4:**
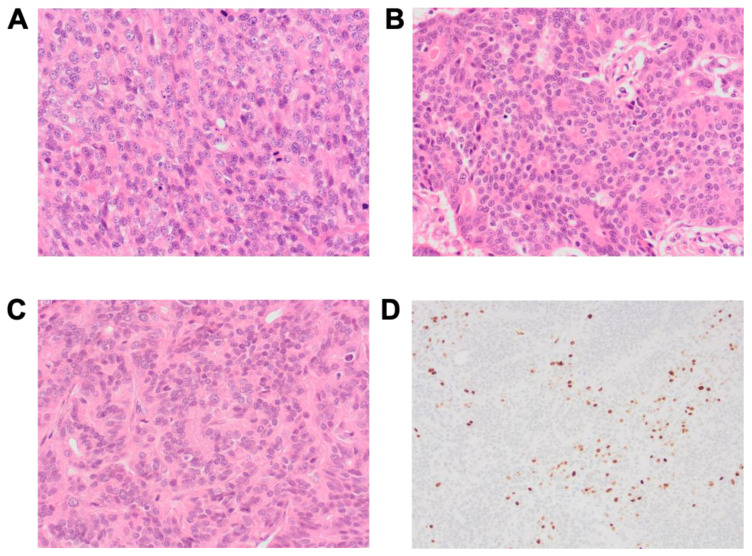
Selected histopathological features of AGASAC lesions. (**A**) Solid pattern: densely packed sheets of neoplastic epithelial cells and a minimal amount of fibrovascular stroma. (**B**). Rosette pattern: neoplastic epithelial cells radially arranged around a small amount of eosinophilic secretion. (**C**). Tubular pattern: neoplastic cells form tubular structures. (**A**–**C**). Haematoxylin and eosin stain magnification ×400. (**D**). Ki67 immunolabelling, Ki67% 54.7, magnification ×200.

The predominant histological pattern was assessed [36], and 23 (23/49; 47%) had a solid pattern, 17 (17/49; 35%) had a rosette pattern, and 9 (9/49; 18%) had a tubular pattern (Figure 4). Intra-tumour necrosis was present in 21 cases (21/49; 43%), and 18 cases (18/49; 37%) had histological evidence of lymphovascular invasion. Assessment of Ki67 immunolabelling was performed in forty-six cases; the remaining three cases had technical issues that prevented assessment. The median Ki67% was 7.75%, with a range of 0−54.7% (Supplementary Materials, Figure S2A). The optimal prognostic threshold for Ki67% was defined as 6.1%. Ki67% and mitotic count showed no correlation in our data (Supplementary Materials, Figure S2B). 

**Figure 5:**
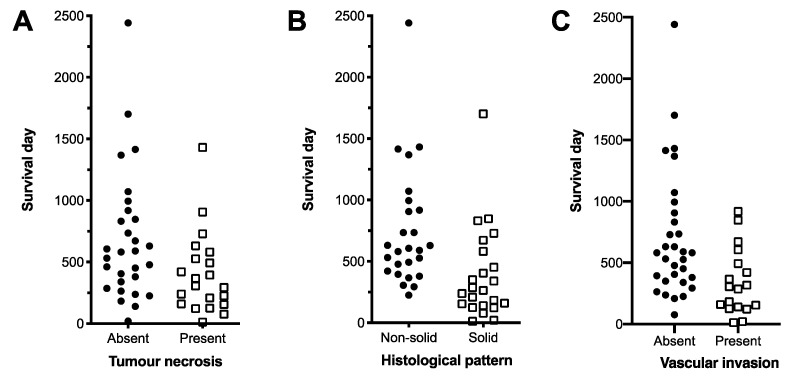
Histopathological variables found to be independent risk factors for poor survival in dogs with AGASAC. (**A**). Tumour necrosis. (**B**). Predominant histological pattern. (**C**). Histological evidence of vascular invasion.

The following histological parameters of the primary tumour were included alongside Ki67 in the global model: tumour necrosis, tumour histological pattern, and evidence of vascular invasion. Ki67% was eliminated from the model during optimisation. The optimised model revealed that a solid histological pattern, necrosis (both in the primary tumour), and vascular invasion were significant independent risk factors of poor survival (Table 6 and Figure 5, Supplementary Materials Table S4).

The parameters of tumour necrosis, histological pattern, and vascular invasion were combined to produce a histopathological algorithm, generating estimated survival times for dogs with AGASAC (Table 7).

**Figure 6:**
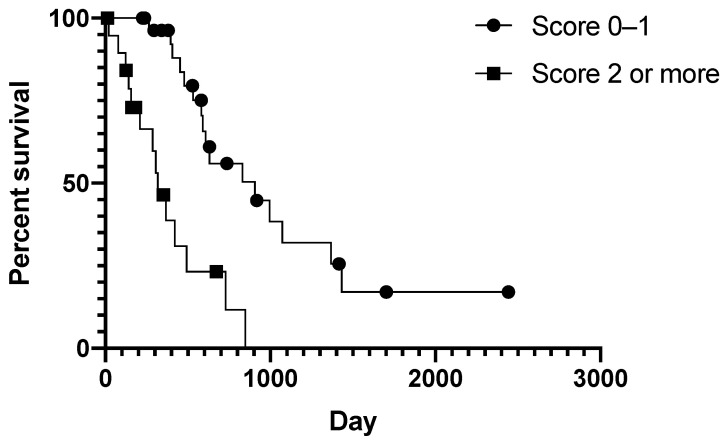
Histopathological algorithm can be used to group AGASAC patients with favourable or worse survival. Kaplan–Meier plot showing cases stratified by the histopathological grading system. Log-rank *p* = 0.0001. Symbols indicate censored cases.

Cases that scored 0−1 (29/49; 59%) had an MST of 906 days, significantly longer than the MST of 318 days for cases that had a score of 2 or more (20/49; 41%) (*p* < 0.0001 Log-rank; Figure 6). The score of 2 or more was associated with a hazard ratio of 4.792, a significant increase in the hazard of death compared to a score of 0−1 (*p* = 0.001 Cox regression; Supplementary Materials Table S5). The algorithm, therefore, was able to stratify the AGASAC cases into two groups with significantly different MST and risk of death.

## 4. Discussion

This study identifies clinical and histopathological features of 81 AGASAC patients that were independent risk factors for decreased survival or PFI. In this paper, we focused on creating a stringent statistical methodology, similar to that used in human oncology, to produce robust results that maximised translational relevance [37]. Careful consideration was given to the number of variables that could be robustly evaluated with the dataset, and variable selection followed recommendations for human medical fields [38]. 

Tumour diameter over 2 cm, lymph node diameter grouped into three tiers by the thresholds of 1.6 cm and 5 cm, and surgical management and/or use of radiotherapy were identified as independent clinical variables associated with survival, irrespective of tumour stage. Tumour diameter over 1.3 cm and presence of distant metastasis were identified as independent clinical variables associated with shorter progression-free intervals. Histological evidence of necrosis, solid histological pattern, and vascular invasion were independent histological variables associated with survival. These data were used to create algorithms based on clinical features and treatment options, or histological features, that significantly influence survival, in order to deliver more accurate estimates of individual survival times. The clinical algorithm utilised staging information, whilst the histological algorithm utilised information from primary tumour tissue. This distinction acknowledges that some AGASAC cases are initially cytologically diagnosed, resulting in clinical staging preceding surgical excision, or that histopathology may be unavailable in cases not managed with surgery. Conversely, in some situations, surgical removal/sampling with histopathology may occur in the absence of full clinical staging.

This paper is the first to use a multivariate model stratified by clinical stage to investigate the effect of clinical parameters on survival. It has been postulated that some effect of tumour size and lymph node size upon survival is often, in part, related to a potential association between larger tumour or lymph node size and increased stage of disease [7,17]. However, in this study, tumour and lymph node size are consistent independent risk factors of decreased survival irrespective of tumour stage. A tumour diameter of 2 cm was identified as the optimum threshold in this study. Previously reported thresholds of tumour size with prognostic value were a diameter of 2.5 cm [7,23] and a volume of 10 cm^2^ (approximate diameter of 3.6 cm) [2]. For studies that use unstandardised methods of determining threshold points, this level of concordance is encouraging. For lymph node size, a two-tier threshold of 1.6 cm and 5 cm was the most discriminatory in these data. The higher threshold of 5 cm is similar to the 4.5 cm threshold previously reported [7], but our data also identified an additional threshold of 1.6 cm, below which cases exhibit a trend for long survival times. Dogs with large primary or lymph node tumour volume, irrespective of distant metastatic disease, can have urofaecal obstruction that may progress to death or euthanasia [5]. In this study, the majority of cases with symptoms of urofeacal obstruction had a tumour diameter over 2 cm and/or lymph node diameter over 5 cm, respectively. We hypothesise that the mass effect of locoregional disease and its sequelae contribute to the association of tumour and lymph node size and survival in our study. 

Successful management of the primary tumour and metastatic lesions, primarily through surgery, can lead to improved survival [2,12,20,21]. Our study demonstrates that locoregional management with surgery and/or radiotherapy can result in significantly longer MST for patients with nodal or distant metastasis when compared to those treated with medical treatment alone or with no treatment. At the study institution, surgery was the predominant form of aggressive locoregional control and was widely offered to patients due to limited access to radiotherapy geographically. This afforded a unique opportunity to assess surgery as a tool for early debulking therapy and interventional palliation of advanced-stage patients that may have otherwise undergone more conservative non-surgical treatment if radiotherapy was readily available. It also explored the impact of locoregional therapy in survival for those patients with distant metastasis when medical management was insufficient to acutely alleviate obstructive symptoms. Our extensive use of CT imaging supported surgical planning, and in our patients, surgery was shown to be safe and effective with a low surgical mortality rate (3%). Tumour or lymph node size was not correlated to the type of treatment received, and therefore, the volume of local disease did not influence patient management. The uncoupling of the clinical stage from the patient outcome is similar to that observed in canine thyroid carcinoma, where distant metastasis has not been a reliable determinant of survival [39,40]. 

The prognostic relevance of tumour and lymph node size is recognised in the current clinical grading system [7], but suggested treatment options are stage-dependent. We suggest an alternative algorithm that places less importance on distant metastasis and instead focuses on the extent, as measured by size, of locoregional disease and its proposed treatment. By using a cumulative score, the algorithm also allows the treatment type to mitigate the effects of presenting tumour and lymph node size, further underscoring the benefits of locoregional control regardless of the original tumour and lymph node size.

In our study population, the incidence of hypercalcaemia was 23%. This study identified a positive association between hypercalcaemia and lymph node size, suggesting that the presence of hypercalcaemia may be related to metastatic volume. Differential expression of parathyroid-hormone-related polypeptide between primary and metastatic lesions has been documented in human mammary carcinoma [41,42] and may suggest a potential mechanism for the association in AGASAC cases. Hypercalcaemia can result in renal damage and other complications, potentially affecting mortality. However, similar to a previous study [15], we found no significant association with survival. It is generally expected that tumour remission will result in the resolution of paraneoplastic hypercalcaemia, and the majority of hypercalcaemic patients in this study were managed surgically, either alone or with adjunctive treatments. We hypothesise that aggressive locoregional management in this study contributed to reducing hypercalcaemia and its sequelae and may explain why hypercalcaemia was not found to be a significant risk factor in this study.

Our study identified tumour size and the presence of distant metastasis as independent significant risk factors in the first PFI. Tumours over 1.3 cm had an almost six-fold increase in the relative risk of a decreased PFI, compared to tumours equal to or less than 1.3 cm. These data are supportive of previously published results in which tumour size influenced the first PFI [15]. The effect of distant metastasis as an independent risk factor for decreased first PFI could be due to the presence of circulating and pre-metastatic seeding of neoplastic cells. Additionally, cases with distant metastasis may be more likely to have an increased frequency of re-examination, and therefore may experience earlier detection of disease progression.

With regard to histopathological features, we identified necrosis, a solid histological pattern, and vascular invasion as independent risk factors of poor survival in multivariate analysis. A solid histological pattern has previously been found to correlate with poor survival on univariate analysis. [11,16,30] Our findings support the hypothesis that loss of the original glandular tissue architecture suggests a loss of cellular differentiation and a more aggressive biological behaviour. Previous evidence also supports an association between decreased survival and the presence of necrosis or vascular invasion [16,30]. We evaluated our hypothesis that two or more of these factors was a more discriminating threshold to differentiate between less favourable and favourable survival times. To achieve this, we combined the independently significant variables into an algorithm based on the presence of two or more of the independently significant variables. Confirming our hypothesis, the algorithm was able to stratify cases into two groups with a significantly different MST and hazard ratio. Using the methodology applied here, Ki67% was not found to be useful in predicting overall survival, a finding consistent with studies [17,30]. Furthermore, Ki67% and the mitotic count showed no correlation in our data.

A limitation of our study is that the retrospective nature resulted in varied patient management, an output of diverse clinical, financial, and ethical considerations. The statistical approach was designed to accommodate this variability, but a subsequent multi-centre study using standardised protocols would be useful to further investigate the findings presented here.

## 5. Conclusions

In this paper, we retrospectively reviewed clinical and histological data from a population of 81 dogs with AGASAC. Tumour and lymph node size, surgical management, and/or radiotherapy were identified as independent clinical variables associated with survival. Tumour size and presence of distant metastasis were identified as independent clinical variables associated with the first progression-free interval. From the histological data, the presence of necrosis, solid histological pattern, and vascular invasion were independent histological variables associated with decreased survival. The independent variables associated with prognosis were incorporated into prognostic algorithms either for use at the clinical staging of AGASAC patients or at histological diagnosis to predict survival. The use of these algorithms, with case-specific adaptations, will aid clinicians and owners, at the time of clinical staging or histological diagnosis, in therapeutic decision making and to project individual outcomes.

## Figures and Tables

**Table 1:** Signalment and clinical signs of study population.

Parameter	Number
Sex	54 males
	27 females
	2:1 male:female
Age	9.7 years mean
	5−15 years interquartile range
Breed most affected	Cocker spaniel n = 30
	Labrador retriever n = 12
	Cross-breed n = 11
Primary clinical sign	Owner-detected perianal mass n = 40
	Incidental finding of perianal mass n = 17
	Dysuria/tenesmus n = 13
	Clinical signs of hypercalcaemia
Mass location	Right anal sac n = 41
	Left anal sac n = 34
	Bilateral n = 6
Sublumbar lymphadenopathy on rectal palpation	Present n = 30
	Absent n = 51

**Table 2:** Model generation for the effect of clinical variables on survival stratified by clinical stage.

Parameter	Groups	No.	MST ^1^	Hazard Ratio	*p* ^2^
Tumour diameter	≤2 cm	35	678	1.983	0.048 *
	>2 cm	42	360		
Lymph node diameter	≤1.6 cm	39	631	3.053	0.001 **
	1.6 < x ≤ 5 cm	24	493		
	>5 cm	18	135		
Surgery	No	18	137	0.332	0.004 **
	Yes	63	531		
Radiotherapy	No	73	452	0.307	0.014 *
	Yes	8	995		
Medical therapy	No	49	452	Eliminated	
	Yes	32	461		
Calcium status	Normal	58	497	Eliminated	
	Increased	19	286		

^1^ Median survival time. ^2^ Based on 999 bootstrap samples. * *p* < 0.05. ** *p* < 0.005

**Table 3:** Median survival times of dogs with AGASAC stratified by clinical stage and treatment.

Clinical Stage	Median Survival Time (days)	
	No Surgery or Radiotherapy	Surgery and/or Radiotherapy
Primary disease only	476	776
Locoregional nodal metastasis	360 ^1^	493 ^1^
Distant metastasis	15 ^1^	421 ^1^

^1^ Log-rank *p* < 0.05.

**Table 4:** Algorithm to predict survival time for dogs with AGASAC based on staging data and proposed treatment.

Parameter	Group	Score
Tumour diameter cm	≤2	0
	>2	1
Lymph node diameter cm	≤1.6	0
	1.6 < x ≤ 5	1
	>5	2
Treatment	Surgery and/or radiotherapy	0
	Neither surgery nor radiotherapy	1

**Table 5:** Optimised multivariate model for the effect of clinical variables on progression-free interval.

Parameter	Groups	No.	MST ^1^	Hazard Ratio	*p* ^2^
Tumour diameter	≤1.3 cm	9	445	5.948	0.006 **
	>1.3 cm	26	230		
Distant metastasis	No	29	252	5.708	0.001 **
	Yes	8	133		
Lymph node size	≤3.8 cm	27	230	0.385	0.053
	>3.8 cm	10	258		
Calcium status	Normal	26	239	Eliminated	
	Increased	8	275		

^1^ Median survival time. ^2^ Based on 998 bootstrap samples. ** *p* < 0.005

**Table 6:** Model generation for histological variables’ effects on survival.

Parameter	Groups	No.	MST ^1^	Hazard Ratio	*p* ^2^
Necrosis	None	29	831	2.984	0.008 **
	Present (any amount)	21	421		
Predominant histological pattern	Non-solid	26	631	2.63	0.04 *
	Solid	23	452		
Vascular invasion	None	31	831	2.794	0.02 *
	Present	18	367		
Ki67%	6.1	21	607	Eliminated	
	>6.1	25	590		

^1^ Median survival time. ^2^ Based on 1000 bootstrap samples. * *p* < 0.05. ** *p* < 0.005

**Table 7:** Histopathological algorithm to predict survival for dogs with AGASAC.

Parameter	Group	Score
Tumour necrosis	None	0
	Present	1
Predominant histological pattern	Rosette or tubular	0
	Solid	1
Vascular invasion	None	0
	Present	1

## Data Availability

The data that support the findings of this study are available in Dryad [data citation, doi:10.5061/dryad.x95x69pgx].

## Supplementary Materials

The following are available online at https://www.mdpi.com/article/10.3390/ani11113327/s1, Table S1: Model generation for the effect of clinical variables on survival stratified by clinical stage, Figure S1: Tumour and lymph node size stratified by treatment received, Table S2: Log-rank and Cox univariate analysis of the clinical algorithm for survival, Table S3: Model generation for the effect of clinical variables on progression-free interval, Figure S2: (a) Ki67% positive cells of primary AGASAC tumours from 46 dogs. (b) Relationship between the Ki67% and mitotic count of individual primary tumours, Table S4: Model generation for histological variables effect upon survival, Table S5: Log-rank and Cox univariate analysis of the prognostic histopathological algorithm.

## Appendix A

Surgical complications reported in dogs following surgical treatment for AGASAC-related disease

Surgical complications included haematoma formation, rectal tearing, dehiscence, post-operative infection, haemorrhage requiring blood transfusion, septic peritonitis, acute renal failure secondary to post-operative pyelonephritis, hypocalcaemia, neurologic signs (sciatic neuropraxia, paraparesis, urinary incontinence), and peri-surgical identification of inoperable disease. Two dogs experienced fatalities, one from non-resectable tumour invasion into the caudal vena cava and aorta and bladder necrosis, and one from cardiorespiratory arrest secondary to suspected systemic inflammatory reaction syndrome (SIRS).

## Appendix B

Radiation therapy for cases of canine AGASAC

Radiation therapy was used in eight dogs (10%), most commonly as an adjuvant treatment to surgery and as palliative therapy in one case. Four dogs received a hyperfractionated protocol after anal sacculectomy and lymphadenectomy. The prescribed dose was 45–48 Gy delivered between 16 to 20 daily fractions given on 5 consecutive days. Hypofractionated radiotherapy using a Quad Shot protocol designated by the radiation oncologist was used in three dogs, which included one dog with a non-resectable primary tumour. The protocol used a prescribed dose of 3.75 to 4 Gy delivered in four fractions over two days and repeated at 21–28 days intervals for 12 fractions. Unacceptable adverse effects (severe diarrhoea, nausea, anorexia, grade 4 neutropaenia) occurred in one dog during the first Quad Shot cycle, and the protocol was subsequently altered (five daily fractions, repeated once in 28 days); mitoxantrone was given concurrently in this case. One dog received radiation treatment following anal sacculectomy (protocol not listed).

## Appendix C

Medical therapy used in treatment for cases of canine AGASAC

Thirty-two dogs (40%) received systemic medical therapy; 20 of these cases also underwent surgery prior. Six dogs received radiation treatment concurrently with medical therapy, either as part of the initial post-operative tumour management (four dogs) or as a form of rescue treatment (two dogs). Additionally, rescue medical therapy was used in 17 dogs. The type of medical therapy was at the discretion of the attending clinician and included carboplatin (n = 12), melphalan (n = 10), metronomic cyclophosphamide (n = 10), mitoxantrone (n = 4), toceranib (n = 16), carboplatin + toceranib (n = 2), metronomic chlorambucil (n = 6), metronomic cyclophosphamide + toceranib (n = 6), doxorubicin (n = 1), masitinib (n = 1), metronomic cyclophosphamide + pamidronate (n = 1), masitinib + metronomic chlorambucil (n = 1), and melphalan + artemisinin (n = 1). Fifty-four dogs (67%) received COX-2 inhibitors. Chemotherapy-associated toxicity was reported in 18 dogs (56%), leading to a treatment change in 14 (44%); these included thrombocytopaenia (n = 9), neutropaenia (n = 6), vomiting/diarrhoea (n = 11), and anorexia/nausea (n = 5). Grade III or IV haematologic toxicities were associated with the use of melphalan (thrombocytopaenia, n = 2), carboplatin (neutropaenia, n = 1), and mitoxantrone (neutropaenia, n = 1). Palladia use was associated with grade 2 ALT increase (n = 1), hypertension (n = 1), and grade 3 proteinuria (n = 3). Sterile haemorrhagic cystitis following cyclophosphamide use was documented in four dogs with compatible clinical signs and a negative urine culture. The median number of medical agents used was two (IQ range 0–6).
