# Supplementary material for: A Retrospective Study of Clinical and Histopathological Features of 81 Cases of Canine Apocrine Gland Adenocarcinoma of the Anal Sac: Independent Clinical and Histopathological Risk Factors Associated with Outcome
Source: Animals (Basel). 2021 Nov 22;11(11):3327. doi: 10.3390/ani11113327 (PMC8614406; doi:10.3390/ani11113327)
Supplement: Supplementary file 1 [file animals-11-03327-s001.zip › animals-1455113-supplementary.pdf]

SUPPLEMENTARY MATERIALS

TABLE S1. Model generation for the effect of clinical variables on survival stratified by clinical stage.

Parameter	Groups	No.	MST †	95%CI‡	Global model Strata = Clinical stage				Optimised model Strata = Clinical stage			
					B§¶	HR«	95%CI	p¶	B§¶	HR«	95%CI	p»
Tumour size	≤2 cm<br>>2 cm	35<br>42	678<br>360	336–1020<br>241–479	0.649	1.914	0.952–3.849	0.091	0.675	1.963	1.013–3.806	0.048*
Lymph node size	≤1.6 cm<br>>1.6– ≤5 cm<br>>5 cm	39<br>24<br>18	631<br>493<br>135	312–950<br>442–544<br>8–258	1.058	2.879	1.549–5.352	0.001	1.116	3.053	1.712–5.446	0.001**
Surgery	No<br>Yes	18<br>63	137<br>531	0–528<br>389–673	-1.023	0.359	0.166–0.776	0.025	-1.103	0.332	0.161–0.685	0.004**
Radiotherapy	No<br>yes	73<br>8	452<br>995	378–526<br>686–1304	-1.208	0.299	0.092–0.967	0.018	-1.18	0.307	0.099–0.949	0.014*
Medical therapy	No<br>Yes	49<br>32	452<br>461	324–580<br>416–506	-0.043	0.958	0.503–1.823	0.906	eliminated			
Calcium status	Normal<br>Increased	58<br>19	497<br>286	412–582<br>145–427	0.341	1.406	0.642–3.079	0.482	eliminated			

†Median survival time.

‡95% confidence interval.

§B coefficient of Cox regression.

¶Unless otherwise noted, bootstrap results are based on 1000 bootstrap samples.

«Hazard ratio.

»based on 999 bootstrap samples.

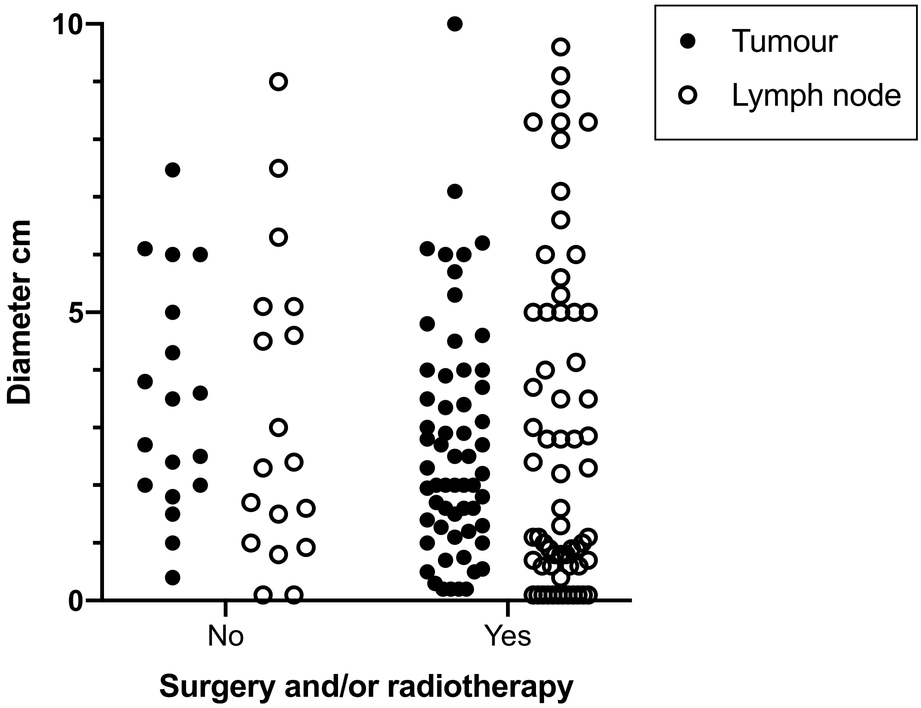

FIGURE S1. Tumour and lymph node size stratified by treatment received.

TABLE S2. Log-rank and Cox univariate analysis of the clinical algorithm for survival.

Score	Number	MST†	95%CI‡	Log-rank p	B§¶	HR«	95%CI‡	Cox p¶
0	15	1072	668−1476	<0.0001 ***	1.351	3.86	2.368–6.29	0.001**
1	29	590	372−808					

2 or more	35	237	22−452					

†Median survival time.

‡95% confidence interval.

§B coefficient of Cox regression.

¶Unless otherwise noted, bootstrap results are based on 1000 bootstrap samples.

«Hazard ratio.

TABLE S3. Model generation for the effect of clinical variables on progression-free interval.

Parameter	Groups	No.	MPFI †	95%CI ‡	Global model				Optimised model			
					B§¶	HR«	95%CI ‡	p¶	B§¶	HR«	95%CI ‡	p‹
Tumour size	≤1.3 cm<br>>1.3 cm	9<br>26	445<br>230	188–702<br>164–296	1.947	7.008	2.25–21.826	0.006	1.783	5.948	2.062–17.161	0.006 **
Distant metastasis	No »<br>Yes	29<br>8	252<br>133	223–281<br>23–243	1.578	4.844	1.773–13.236	0.002	1.742	5.708	2.093–15.567	0.001 **
Lymph node size	≤3.8 cm<br>>3.8 cm	27<br>10	230<br>258	223–281<br>23–243	-0.763	0.466	0.173–1.259	0.123	-0.954	0.385	0.155–0.96	0.053
Calcium status	Normal<br>Increased	26<br>8	239<br>275	224–254<br>174–376	-0.525	0.592	0.203–1.723	0.338				

†Median first progression-free interval

‡95% confidence interval.

§B coefficient of Cox regression.

¶Unless otherwise noted, bootstrap results are based on 1000 bootstrap samples.

«Hazard ratio.

»Locoregional spread or primary disease only.

‹Based on 998 samples.

FIGURE S2. (a) Ki67% positive cells of primary AGASAC tumours from 46 dogs. (b) Relationship between the Ki67% and mitotic count of individual primary tumours.

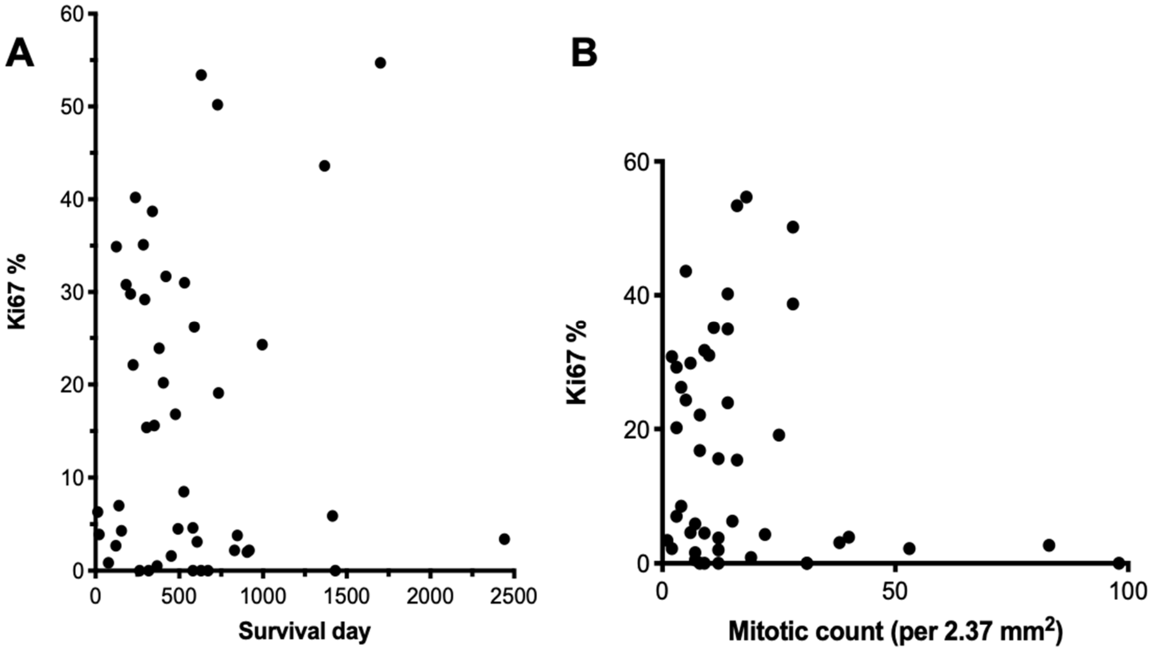

TABLE S4. Model generation for histological variables effect upon survival.

Parameter	Groups	No.	MST †	95%CI‡	Global model				Optimised model			
					B§¶	HR«	95%CI ‡	p¶	B§¶	HR«	95%CI‡	p¶
Necrosis	None<br>Present	28<br>21	831<br>421	498–1163<br>268–574	1.094	2.987	1.291–6.913	0.014	1.093	2.984	1.336–6.662	0.008**

Predominant histological pattern	Non-solid<br>Solid	26<br>23	631<br>452	85–1177<br>179–725	0.986	2.68	1.177–6.103	0.068	0.967	2.63	1.194–5.793	0.016*
Vascular invasion	None<br>Present	31<br>18	831<br>367	433–1229<br>233–501	1.252	3.497	1.408–8.683	0.011	1.027	2.794	1.251–6.242	0.012*
Ki67%	≤6.1<br>>6.1	21<br>25	607<br>590	178–1036<br>419–761	0.382	1.465	0.623–3.443	0.445	eliminated			

†Median survival time.

‡95% confidence interval.

§B coefficient of Cox regression.

¶Unless otherwise noted, bootstrap results are based on 1000 bootstrap samples.

«Hazard ratio.

TABLE S5. Log-rank and Cox univariate analysis of the histopathological prognostic algorithm.

Score	No.	MST†	95%CI‡	Log-rank p	B§¶	HR«	95%CI‡	Cox p¶
Score 0−1	29	906	374−1438	<0.0001****	1.567	4.792	2.127−10.796	0.001**
Score 2 or more	20	318	224−412					

†Median survival time.

‡95% confidence interval.

§B coefficient of Cox regression.

¶Unless otherwise noted, bootstrap results are based on 1000 bootstrap samples.

«Hazard ratio.
